# Gallium Nitride Based Electrode for High‐Temperature Supercapacitors

**DOI:** 10.1002/advs.202300780

**Published:** 2023-03-25

**Authors:** Songyang Lv, Shouzhi Wang, Lili Li, Shoutian Xie, Jiaoxian Yu, Yueyao Zhong, Guodong Wang, Chang Liang, Xiangang Xu, Lei Zhang

**Affiliations:** ^1^ Institute of Novel Semiconductors State Key Lab of Crystal Materials Shandong University Jinan 250100 P. R. China; ^2^ Shenzhen Research Institute Shandong University Shenzhen 518000 P. R. China; ^3^ Institute of Crystal Materials State Key Lab of Crystal Materials Shandong University Jinan 250100 P. R. China; ^4^ School of Public Administration Shandong Normal University Jinan 250100 P. R. China; ^5^ Key Laboratory of Processing and Testing Technology of Glass & Functional Ceramics of Shandong Province School of Materials Science and Engineering Qilu University of Technology (Shandong Academy of Sciences) Jinan 250353 P. R. China; ^6^ School of Materials Science and Engineering Shandong Jianzhu University Jinan 250100 P. R. China

**Keywords:** density functional theory, heterostructures, high temperatures, porous GaN, supercapacitors

## Abstract

Gallium nitride (GaN) single crystal, as the representative of wide‐band semiconductors, has great prospects for high‐temperature energy storage, of its splendid power output, robust temperature stability, and superior carrier mobility. Nonetheless, it is an essential challenge for GaN‐based devices to improve energy storage. Herein, an innovative strategy is proposed by constructing GaN/Nickel cobalt oxygen (NiCoO_2_ ）heterostructure for enhanced supercapacitors (SCs). Benefiting from the synergy effect between the porous GaN network as a highly conductive skeleton and the NiCoO_2_ with massive active sites. The GaN/NiCoO_2_ heterostructure‐based SCs with ion liquids electrolyte are assembled and delivered an impressive energy density of 15.2 µWh cm^−2^ and power density, as well as superior service life at 130 °C. The theoretical calculation further explains that the reason for the energy storage enhancement of the GaN/NiCoO_2_ is due to the presence of the built‐in electric fields. This work offers a novel perspective for meeting the practical application of GaN‐based energy storage devices with exceptional performance capable of operation under high‐temperature environments.

## Graphical abstract

The porous GaN/NiCoO_2_ heterostructure is designed as an advanced electrode and assembled SCs combine with ionic liquids electrolytes, and the device displays excellent performance at 130 °C. The theoretical calculation further explained that the reason for the energy storage enhancement of the GaN/NiCoO_2_ is due to the presence of the built‐in electric fields.

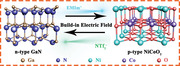

## 1 Introduction

Demand for the availability of excellent performance, cost effective, and environmentally benign energy storage systems increase with the widespread rise of electric vehicles and portable electronics.^[^ ^1^ ^]^ The large‐scale application of lithium‐ion batteries has evoked a crisis of diminishing lithium reserves.^[^ ^2^ ^]^ Supercapacitors (SCs) have captured numerous research attention as merits that their higher power output, faster energy harvest, and ultra‐longer cycle lifespan.^[^ ^3^ ^]^ In light of those, SCs generally considered as integration and/or supplement for batteries in certain energy storage markets.^[^ ^4^ ^]^ Especially in high‐temperature environments, such as the engine cover of hybrid electric vehicles can exceed 140 °C and the locate temperature of specific tasks (subsurface exploration, medical rescue, and military) may be more than 250 °C.^[^ ^5^ ^]^ SCs have become mainstream applications since most electrolytes in commercial organic batteries with boiling points below 80 °C lead to the instability of devices.^[^ ^6^ ^]^ Therefore, it is necessary to develop reliable, safe, and prominent performance SCs that operate under high‐temperature conditions. Most discussed mitigating thermal damage in energy storage systems have concentrated on additional heat sinks, which lead to extra cost and spatial inefficiency.^[^ ^7^ ^]^ The rational design of SCs is crucial for outstanding performance and safety at high‐temperature conditions. This is expected to meet the requirements of the new generation of energy storage systems, although it presents huge challenges.

The electrode material is the essential factor, which affects the whole performance of SCs under high‐temperature environments.^[^ ^8^ ^]^ Wide band gap semiconductors, their properties are satisfactory in a variety of applications including integrated circuits, energy conversion, and so on.^[^ ^9^ ^]^ Up to now, third‐generation semiconductor materials have proven to be competitive candidates within the range of high‐temperature electrochemical storage. For instance, Li.^[^ ^10^ ^]^ designed and prepared the SCs of silicon carbide (SiC) nanochannel arrays electrode, which exhibited impressive performance of 14.8 mF cm^−2^ at 60 °C and over 95% capacitance retention was achieved; Lv^[^ ^11^ ^]^ fabricated few‐layered gallium nitride (GaN) crystal and the assembled device with the capacitance of 33.7 mF cm^−2^ that operate at 100 °C; Li^[^ ^12^ ^]^ manufactured SCs based on SiC nanowires as an electrode, delivering capacitance of 13.0 mF cm^−2^ under 100 °C and 75.5% of initial capacitance was maintained after 10 000 cycles. Nonetheless, it is a common conundrum to further improve the capacity of the device for exploiting the advantages of wide band gap semiconductor‐based SCs in high temperatures.

Massive attempts have been focused on broadening the voltage window and ameliorating the capacitance of electrodes to enhance the energy storage of SCs.^[^ ^13^ ^]^ On the one hand, an effective approach for boosting the capacitance of the electrode is to combine the strong point of excellent electrical conductivity and plentiful reactive site materials for building up a heterostructure electrode.^[^ ^14^ ^]^ For example, Sun^[^ ^15^ ^]^ constructed an EG@SiC Schottky junction, which successfully facilitated Li ions storage; Li^[^ ^16^ ^]^ reported a SiC@C nanowire arrays electrode and its specific capacitance exceeded 700% that of a pure SiC species electrode. Whereas, as one of the representatives of the wide band gap semiconductor, GaN‐based SCs with admirable capacity storage are more sparsely reported. In this regard, the porous n‐type GaN crystal with a highly long‐range ordered structure, where provide a larger specific surface area and retain its inherent features such as splendid power output, robust temperature stability, and superior carrier mobility,^[^ ^17^ ^]^ which is considered a revolutionary “skeleton” applied in high‐temperature energy storage application.^[^ ^18^ ^]^ The binary transition metal oxides (NiCoO_2_, abbreviated as NCO) that ideal theoretical capacity and variable valence, which is desirable directly grafted onto porous GaN crystal “skeleton” to set up GaN‐based integrated electrode. The porous GaN served as a conductive substrate conducive to the uniform distribution of active materials, which avoided the issue of NCO severe aggregation.^[^ ^19^ ^]^ The NCO nanosheets (NSs) provide abundant active sites, which display remarkable electrochemical activity. On the other hand, selecting ionic liquids (ILs) with thermal stability, nonflammability, and a wide range of operating voltages as electrolytes,^[^ ^20^ ^]^ which is another crucial route to improve the energy storage of the GaN‐based devices without sacrificing the power density at high‐temperature environment.

In this work, a porous GaN/NCO heterostructure electrode is designed and fabricated by a simple and efficient strategy. Afterward, the device is assembled via a porous GaN‐based heterostructure electrode combined with ILs and exhibits amazing properties in high‐temperature environment. Obviously, the ingenious structure design of the intense bonding between porous GaN substrate and NCO NSs is beneficial to achieve synergy. The interconnected pore channel not only reduces the route of ions/electrons diffusion and migration but also effectively alleviates volume expansion under strong current shock for enhancing the stability of the device. Importantly, the GaN/NCO heterostructure increases the specific surface area, and active sites and plays a dominant site to strengthen the adsorption strength of ILs for improving the energy capture capability of the device. This study provides an innovative strategy and opens up broader application prospects for GaN crystal application in high‐temperature conditions.

## 2 Results and Discussion

**Figure 1:**
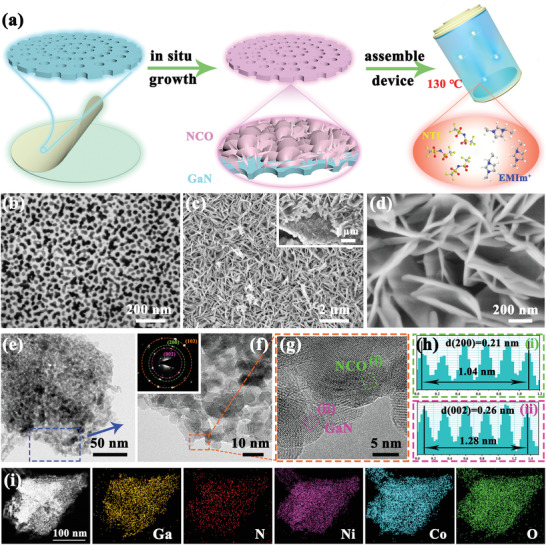
a) Schematic illustration synthesis process of the GaN/NCO heterostructure. SEM images of porous GaN (b), porous GaN/NCO heterostructure (c), and the magnified image (d) respectively; e,f) TEM images and corresponding SAED pattern; g) HRTEM image; h) relevant linear profiles defined lattice fringe spacing; i) element (Ga, N, Ni, Co, O) mapping images of GaN/NCO heterostructure respectively.

The GaN‐based heterostructure with a range of different Ga/M (M=Ni or Co) ratios is synthesized through a facile approach, which is systematically displayed in **Figure** **1**a. The detailed fabrication protocol is described in Experimental Section (Supporting Information). Figure 1a illustrates that interconnected p‐type NCO NSs array in situ growth on an n‐type porous GaN membrane. Briefly, according to previous research results,^[^ ^21^ ^]^ the porous GaN exfoliated from the sapphire substrates is acquired by employing an electrochemistry etching technique regulating the rate‐limiting factor of carriers (Figure S1, Supporting Information). Subsequently, the Ni‐Co precursors are homogeneously formed on the surface of the porous GaN through a one‐step hydrothermal route. Followed by thermal annealing and the architecture is well maintained during the transformation of Ni‐Co precursors to NCO.

The morphology of the porous GaN/NCO heterostructure is characterized by scanning electron microscopy (SEM). Consistent with the mole ratio of each substance, the samples are marked as GaN, GaN/NCO‐1, GaN/NCO‐2, GaN/NCO‐3, and NCO (Experimental Section in Supporting Information). Compared with the pristine GaN wafer, the surface of electrochemistry treatment n‐type GaN membrane behaves evenly distributed pores with a diameter of ≈40 nm, and the pores run through the entire membrane while interdigitating to construct the conductive network structure with a thickness ≈1.64 µm (Figure 1b; Figures S1,S2, Supporting Information). Owing to the electrochemistry etching process, there are numerous defects existed in the porous GaN. These defects can act as key points for the NCO NS's growth and are anchored easily on the porous GaN substrate. The SEM images of GaN/NCO‐1 (Figure S3a,b, Supporting Information) depict that the Ni‐Co source is so deficient that the porous GaN surface is scarcely covered, while the NCO component tends to condense slightly. As the ratio of transition metal source increases, it is observed that the external porous GaN substrate is decorated with NCO NSs without apparent aggregation and collapse after calcination (Figure 1c). In addition, the NCO NSs cross‐link with each other to make up a wall‐like open configuration as in enlarged Figure 1d. The phenomenon of growth may be analogous to the well‐known “house of cards” conformation, which is universally mentioned in two‐dimension NSs with outstanding electrical properties.^[^ ^22^ ^]^ With the proportion of transition metal components further expanded, the accumulation and partial collapse emerge on the surface of porous GaN (Figure S3c,d, Supporting Information). The cluster stacking leads to an invalid connection between porous GaN substrate and active materials, which inevitably increases the resistance of the material and is unfavorable for the electrochemical reaction. Severe self‐aggregation occurs in NCO microspheres constituted of nanoneedles when without GaN substrate is added (Figure S4a–c, Supporting Information).

Transmission electron microscopy (TEM) and high‐resolution (HR) TEM at a series of magnifications are employed to further determine the morphology and microstructure of the GaN/NCO‐2 heterostructure. From the images in Figure 1e,f, it can be clearly indicated that the NCO NSs neatly developed on the porous GaN substrate. In stark contrast, the bare porous GaN presents ultrathin and porous features, which are insinuated by an almost transparent appearance (Figure S2c, Supporting Information). And typical aggregated microspheres appear in NCO without porous GaN substrate, which assembled from nanowires hold an average diameter of belike 40 nm (Figure S4d,e, Supporting Information). The morphologies of all samples correspond to the results of the respective SEM investigations. The selected area electron diffraction (SAED) pattern of the GaN/NCO‐2 heterostructure is collected in the inset of Figure 1f, where the bright diffraction spots implied that the porous GaN‐based heterostructure retains well crystallinity. The shiny spots in the SAED pattern can be indexed to (002) and (103) lattice planes of hexagonal symmetric GaN, as well as, the (200) lattice plane of NiCoO_2_ can also be noticed. The HRTEM (Figure 1g) view of selected regions and the relevant linear profiles (Figure 1h) are recorded in order to define accurate lattice fringe spacing of 0.26 and 0.21 nm. These lattice spacing belong to the (002) plane of the hexagonal GaN single crystal and the (200) plane of the NCO NSs respectively, which are well in agreement with that of an individual substance (Figures S2d,S4f, Supporting Information). The element mapping images (Figure 1i) and the corresponding elemental energy‐dispersive X‐ray (Figure S5, Supporting Information) certificate that the five elements of Ga, N, Ni, Co, and O are uniformly assigned without any other impurity elements. This even dispersion favors synergistic effects between each element.

**Figure 2:**
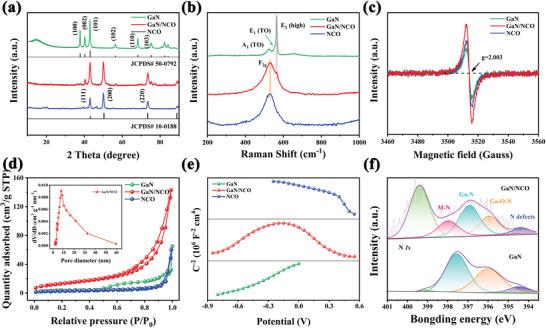
Structural characterization of GaN/NCO‐2 heterostructure. a) XRD pattern; b) Raman spectra; c) EPR patterns; d) N_2_ adsorption‐desorption isotherms with inset showing the pore size distribution plots; e) M–S curve; f) High‐resolution N *1s*.

To characterize the crystallographic phase of the GaN/NCO‐2 heterostructure resultant products, the powder X‐ray diffraction pattern is plotted as shown in **Figure** **2**a. It is inspected that all characteristic peaks of the obtained GaN, GaN/NCO heterostructure, and NCO completely match with the standard card of the GaN (PDF#50‐0792) and the NiCoO_2_ (PDF#10‐0188). This confirms the coexisting of GaN and NCO without whichever contaminants. It is noteworthy that the peak intensity of the GaN in GaN/NCO is slightly weakened, which is attributed to the coverage of NCO NSs.

Raman measure is performed to further detect the structure of the GaN/NCO‐2 heterostructure. In Figure 2b, the E_2_ (high) peak of porous GaN and characteristic signal at 532 cm^−1^ of GaN/NCO‐2 heterostructure are found.^[^ ^23^ ^]^ The coexistence of porous GaN and NCO is proved. Compared with pure porous GaN, the slight shift in the site of E_2_ (high) is the result of internal stress between porous GaN and NCO NSs, which suggested their intense interfacial coupling of them.^[^ ^24^ ^]^ In addition, the Raman spectrum of porous GaN emerged A_1_ (TO) and E_1_ (TO) stretching modes with signal ≈527 and 554 cm^−1^ respectively, which ascribed to the large number of defects introduced in the electrochemistry etching process.^[^ ^25^ ^]^ The Fourier transform infrared spectrum (Figure S6, Supporting Information) of GaN, GaN/NCO‐2 heterostructure and NCO further identify the above conclusions. The stretching mode of Ga—N, M—O, along with M—N is gathered.

Furthermore, the electron paramagnetic resonance (EPR) is adopted to understand the structural defects of the GaN/NCO‐2 heterostructure and porous GaN, NCO for comparison. In Figure 2c, it can be readily found that the center values of symmetrical signals are similar but have different intensities of them. The peak center at a g value of 2.003 and a g factor of 2.004 are related to oxygen vacancies of NCO and nitrogen vacancies of the porous GaN, respectively. In contrast to others, the GaN/NCO‐2 heterostructure exhibits a stronger peak in the EPR diagram, which indicates that more vacancies exist in the heterostructure. The vacancy has an important role in promoting reaction kinetics and regulating the electronic structure thanks to its shallow donor effect.^[^ ^26^ ^]^ Hence, the activity of the electrode is enhanced, which is achieved by defects in the reaction phase.

Aiming to fully comprehend the structural integrity of the GaN/NCO‐2 heterostructure, the N_2_ adsorption‐desorption isotherms curves of all samples are described in Figure 2d. As presented in Figure 2d, the shapes of all lines represent typical IV types that enriched macroporous and mesopores adsorption. In the region where the relative pressure is 0.4–1, an evident type of H_3_ hysteresis loop is able to witness, demonstrating that the mesopores derived from the special structure and hole/slit interconnection of the porous GaN‐based heterostructure. Based on the Brunauer–Emmett–Teller measure, the specific surface area of GaN/NCO‐2 heterostructure is counted to be 49.04 m^2^ g^−1^, which is greater than that of unitary porous GaN (14.02 m^2^ g^−1^) and pure NCO (9.98 m^2^ g^−1^). This conclusion can speculate from the morphological distinction among them. The average pore size distribution parameters and porosities are analyzed by Barret–Joyner–Halenda method. In the inset of Figure 2d, the pore diameter for the GaN/NCO‐2 heterostructure is concentrated at the 15.58 nm, signifying that mesoporous adsorption is dominant. For the sake of comparison, the pore size of porous GaN and NCO is also investigated (Figure S7, Supporting Information).

Additionally, the content ratio of porous GaN and NCO NSs in the GaN/NCO‐2 heterostructure complex is monitored through thermogravimetric analysis (TGA) (Figure S8, Supporting Information). Contrasting the TGA plots of the porous GaN, the considerable weight reduction of the other samples, the result derives from the evaporation of physically adsorbed water and the removal of structure water during the conversion of hydroxyl species to oxides is occurred. According to the date of TGA, it can be calculated that the content of NCO in GaN/NCO‐2 is ≈44.5 wt.%.

The interface interaction between GaN and NCO NSs is also demonstrated by the Mott–Schottky (M–S). In Figure 2e, it is observed that the line with a positive slope of porous GaN as expected, expressing typical n‐type semiconductor features. Meanwhile, NCO is confirmed as a p‐type semiconductor in view of the M–S curves. Interestingly, the curves of the GaN/NCO‐2 exhibit an inverted V‐shape, which directly indicates the heterostructures' existence between GaN and NCO NSs.^[^ ^27^ ^]^ In terms of the M–S formula (Supporting Information), the electron concentration of porous GaN is calculated to be 6.1 × 10^22^ cm^−3^. It is a satisfactory substrate with congenital conductivity for rapid charge transfer.

X‐ray photoelectron spectroscopy (XPS) is carried out to explore the element composition and bonding structure of the GaN/NCO‐2 heterojunction structure. From the survey spectra (Figure S9, Supporting Information), it is disclosed that Ga, N, Ni, Co, and O elements appear in GaN/NCO‐2 heterostructure. The Ga‐N and Ga‐O‐N peak are detected in the Ga *3d* XPS spectrum (Figure S10a, Supporting Information), which is capable of being divided into two deconvoluted peaks position at 19.7 and 20.9 eV, respectively.^[^ ^28^ ^]^ In the O *1s* region of GaN/NCO‐2 heterostructure (Figure S10b, Supporting Information), the XPS spectrum can be annotated at 530.0, 531.7 and 533.3 eV are the symbol of metal‐oxygen species, oxygen vacancy and physical/chemical absorbed oxygen.^[^ ^29^ ^]^ Notably, the occurrence of O element in porous GaN crystal originates from the generation of a small amount of oxygen adsorption and oxygen functional groups at the defects and active site during the EC process.^[^ ^30^ ^]^ More knowledge is gained from the N *1s* spectrum (Figure 2f), where the N *1s* peak of the GaN/NCO‐2 heterostructure can be deconvolved into four pairs center at 397.4, 396.1, 399.3, and 394.5 eV, referring to Ga—N, Ga—O—N, N—H, and N defects in accordance with previous reports.^[^ ^31^, ^32^ ^]^ Interestingly, it is noted that the slight binding energy movement compared with the Ga *3d* and N *1s* of pristine porous GaN. This may be caused by the redistribution in the charge condition through the interfacial phase interaction between porous GaN and NCO NSs.^[^ ^33^ ^]^ By looking closer, a new peak of the GaN/NCO heterostructure has been recognized as M—N bonds,^[^ ^34^ ^]^ which directly certificating the strong interfacial coupling and the formation of the heterostructure between porous GaN and NCO NSs. This conclusion is also consistent with the results of Raman and M–S analysis. Additionally, there are two shakeup satellites and a pair of fitting peaks of Ni *2p* and Co *2p* core‐level spectra (Figure S10c,d, Supporting Information).^[^ ^35^, ^36^ ^]^

**Figure 3:**
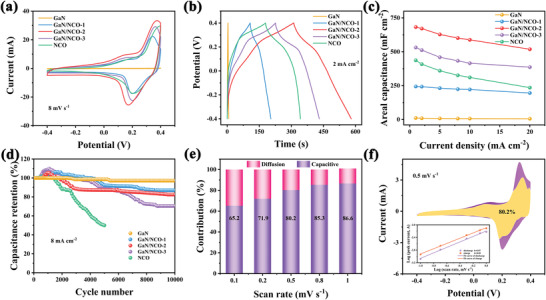
Electrochemical performance of the GaN/NCO heterostructure. CV curves (a) and GCD measures (b); c) the areal capacitance of various current densities; d) the cycling performances; e) normalized contribution ratios of the capacitive process at different scan rates; f) the capacitive contribution as a percentage of total capacitance at 0.5 mV s^−1^ and the relationship between the logarithm of scan rate and peak current.

To determine the effectiveness of the GaN‐based heterostructure design and optimization on electrochemical behavior, the three‐electrode configuration is executed. In **Figure** **3**a, the GaN/NCO‐2 heterostructure electrode holds a quite larger area of CV curve compared to pure porous GaN and NCO, illustrating a remarkably enhanced capacitive performance from the unusual heterostructure architecture and synergy effect of each composition. Nevertheless, it is able to bring about the decline of the CV curve area for GaN/NCO‐3 and NCO when the content of the NCO is further increased. This is probably the result of structural collapse and material accumulation as revealed in SEM images. The CV curves of all samples (except porous GaN) exhibit a couple of redox peaks that are representative of the Faradaic reaction, which is allocated to reversible change of valence state between M^2+^/M^3+^ (M: Ni or Co). Besides, the detailed electrochemical performance of each sample is presented in Figures S11–S15 (Supporting Information). The galvanostatic charge‐discharge (GCD) measurements of all samples at 2 mV s^−1^ are displayed in Figure 3b. The well symmetry of the GCD curves also clarifies the highly reversible features of the redox and the satisfactory coulombic efficiency (≈100%) during repeated charging and discharging. The GCD profiles of the GaN/NCO‐2 heterostructure compound contain a longer discharge period than individual NCOs, which certifies porous GaN beneficial to promote electrochemical activity. Notability, two feature regions are included in the GCD profiles, one with the linear charge‐discharge curves which comply with the surface charge storage process and the other is nonlinear charge‐discharge curves with sloping plateaus that are dependent on redox substances. The specific areal capacitance of the electrodes at various current densities is calculated and the relevant data are plotted in Figure 3c. The capacitance value of the GaN/NCO‐2 heterostructure electrode reaches as high as 683 mF cm^−2^ at 1 mA cm^−2^, which exceeds the rest of the that comparative samples. More importantly, the capacitance retention of 76% for GaN/NCO‐2 heterostructure even upon 20 mA cm^−2^ of current density, confirming the brilliant rate capability and stability of GaN/NCO‐2 hybrid electrode, compared with that of 53.8% for NCO. Obviously, the distinctive GaN‐based heterojunction structure is beneficial to explore more specific surface area and active sites for easier contact with electrolytes to advance capacitance performance, particularly when low current density. The favorable rate performance and conductivity of the porous GaN‐based hybrid material especially at high current density is the result of the porous GaN with inherently excellent carrier mobility and abundant mesopores, which provide transport diffusion channels for ions/electrons.^[^ ^37^ ^]^

The long‐term cycling data of the prepared electrodes at a current density of 8 mA cm^−2^ are gathered in Figure 3d. After 10 000 cycles, the capacitance of the GaN/NCO‐2 heterostructure electrode maintained 82% of the initial capacitance. While the NCO capacity decays the fastest and maintains only 50% after 5 000 cycles, which may be caused by the collapse and accumulation of the structure. A stark contrast, the capacitance retention of the porous GaN electrode is 98%, with hardly any change in capacitance after 10 000 cycles. It obviously manifested that the cycling stability of porous GaN‐based heterostructure dramatically ameliorated by incorporating porous GaN conductive substrate.^[^ ^38^ ^]^ The improvement of cycling performance in the initial stage is attributed to electrode activation.^[^ ^39^ ^]^ In general, the superior electrochemistry performance of the GaN/NCO‐2 heterostructure originates from the synergistic effect of porous GaN and NCO NSs. Electrochemical impedance spectroscopy (EIS) is utilized to understand the transport ion/electron kinetics in the electrode, the Nyquist plots are shown in Figure S16 (Supporting Information). All curves make up of a semicircle where the high‐frequency region is and a linear line where the low‐frequency region is. It can be distinctly found that the GaN/NCO‐2 heterostructure electrode has a smaller radius of the semicircle and a larger straight‐line slope than the NCO electrode, which implies a better charge transfer rate and superior ion diffusion ability.^[^ ^40^ ^]^ In view of the above analysis, it can be speculated that the construction of the porous GaN‐based heterostructure design is a valid strategy to optimize energy storage. As expected, the electrochemical performance exceeds that of most related heterostructure materials reported (Table S1, Supporting Information).

Aiming to thoroughly interpret the mechanism of the charge storage process, the capacitance contribution is calculated by quantitatively distinguishing the percentage of diffusion‐controlled process and capacitance‐controlled process. The results of the GaN/NCO‐2 heterostructure capacitance contribution are summarized in Figure 3e. It can be seen that the reactions from capacitive control dominate the overall current contribution and increased capacitance contribution from 65.2% to 86.6% as the scan rates rise. The capacitive contribution of 80.2% at 0.5 mV s^−1^ is plotted in Figure 3f as a representative calculation. Similarly, this result can also be certificated by discussing the relationship between the peak current and scan speed. The corresponding b value and fitting curves of the GaN/NCO‐2 heterostructure are illustrated in Figure 3f inset. The b value of the anodic peak is 0.87 and the cathodic peak is 0.83 certifying the reaction is a synergism, which relies on surface capacitance and diffusion capacitance as well as the capacitance mainly comes from surface adsorption and pseudocapacitance. It can be reasoned that the heterostructure is beneficial for boosting reaction dynamics and rate performances.

**Figure 4:**
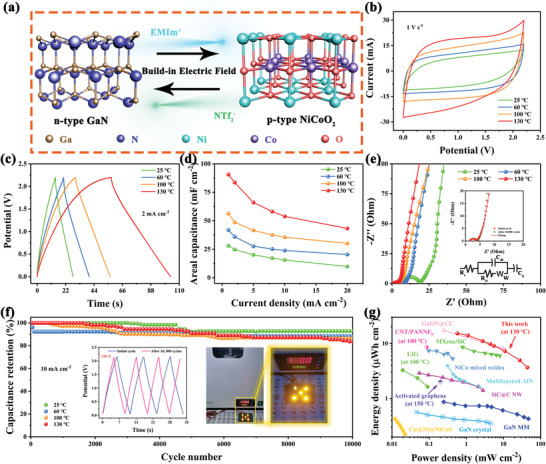
The electrochemical properties of the GaN‐based devices in a high‐temperature environment. a) The schematic illustration of the GaN‐based heterostructure; the CV curves (b) and the GCD curves (c); d) the areal capacitance at various current densities; e) Nyquist plots of the porous GaN‐based heterostructure (the circuit fitting model inset in it); f) the cycling performances, inset exhibited the GCD curves of the before and after 10 000 cycles (left), and the images of commercial LED powered by the fabricated device at 130 °C (right); g) Regine plots in comparison with the relevant SC devices reported in the literature.

**Figure** **4**a schematically illustrates that the GaN‐based heterostructure evolved a built‐in electric field into the electrode, which serves as a key point to enhance the adsorption of electrolyte ions and the electrochemical performance of the device. To assess the energy storage potential of the GaN/NCO heterostructure in a high‐temperature environment, the fresh SCs are assembled with GaN/NCO‐2 heterostructure as electrode and ILs as the electrolyte, which operated over a series of progressively elevate working temperature ranges. The quasi‐rectangular CV curves express common electric double layer capacitors (EDLC) feature and the redox peaks do not appear, which entails the distinguished capacitive behavior under 25–130 °C. And the details of individual temperatures are presented in Figures S17–S20 (Supporting Information). Specifically, even the shape of CV curves is not appreciably distorted when the scan rates are expanded 800 times under 130 °C conditions, implying splendid reversibility and rate capability. The GCD plots of the device under various temperatures at 2 mA cm^−2^ are emerged in Figure 4c. The appearance of quasi‐triangular reveals the ideal capacitive trait, which corresponds to the CV measures. Additionally, the areal‐specific capacitance in the varied temperature and diverse current density is computed (Figure 4d). The acceptable capacitance value of 90.6 mF cm^−2^ is reached at the current density of 1 mA cm^−2^ under the temperature of 130 °C, which is markedly beyond that of 28.4 mF cm^−2^ for 25 °C at the same current density. The remarkable energy storage performance improvement under a high‐temperature environment is primarily sourced from the raised conductivity and reduced viscosity of the ILs.^[^ ^12^ ^]^ This speculation is evidenced by researching EIS of GaN/NCO heterostructure‐based SC with different temperatures. In the Nyquist plot (Figure 4e), the intercept of the real axis where the beginning of the high‐frequency area drops clearly as the temperature goes up, which hinted less internal resistance of active material and electrolyte. Moreover, the interfacial resistance between active material and electrolyte is gradually diminishing, which suggests a faster charge transfer rate and enhanced affinity among them. It can be distinctly verified that the ionic conductivity of IL ions in the device at 130 °C is upgraded compared with the room temperature state. In the zone of the low frequency, the Nyquist plots of all temperatures present almost vertical lines, which acknowledging the desirable capacitive behavior and shorter Warburg district under high temperature facilitate electrolyte ions diffuse into the interior of the electrode. And relevant circuit fitting diagram has been displayed in the inset of Figure 4e and Table S2 (Supporting Information).

The stability is also a pivotal parameter to determine the practical application of the SCs in high‐temperature environments (Figure 4f). Surprisingly, the impressive cycling lifespan is attained and delivers a capacitance retention of 83.6% after 10 000 cycles at 130 °C. The stability of the GaN/NCO heterostructure electrode structure enables the IL‐based device to effectively resist repeated large current shocks at high temperatures for acquiring amazing performance. As is apparent, it is hard to discover the morphology changes of the GaN/NCO‐2 heterostructure electrode from the SEM images (Figure S21, Supporting Information), which is inspected after 10 000 cycles at 130 °C. The GCD curves and EIS measures of the initial and that after 10 000 cycles are compared in the inset of Figure 4f left and Figure 4e, which both further emphasized the long‐term stability of the device in a high temperature of 130 °C. In Figure 4f inset right, it is demonstrated that the porous GaN‐based device is able to power five light‐emitting diodes (LED) with the ″N″ pattern. It verifies the practical application potential of the device that runs at a high‐temperature zone. Apart from that, the long‐term cycling operation is executed as successive temperature changes (Figure S22, Supporting Information). As expected, it exhibits remarkable stability and reversibility. The high‐temperature electrochemical properties of the porous GaN/NCO heterostructures‐based SCs with ILs as electrolytes exceed those related devices recorded in Table S3 (Supporting information). Energy density and power density are required for modern energy storage devices. The porous GaN‐based heterostructure devices deliver a maximum energy density of 15.3 µWh cm^−2^ and maximum power density of 44.0 mW cm^−2^ (Figure 4g), which the number of energies that endow be captured is boosted while hardly any without sacrificing the power density at the high‐temperature environment. Markedly, the devices are overwhelmingly superior to the rest of the relevant SCs reported in the literature, such as MXene@SiC (8.5 µWh cm^−2^, 0.8 mW cm^−2^),^[^ ^41^ ^]^ SiC/C (2.8 µWh cm^−2^, 0.5 mW cm^−2^),^[^ ^16^ ^]^ GaON@CC (8.1 µWh cm^−2^, 10 mW cm^−2^),^[^ ^42^ ^]^ NiCo mixed oxides (7.4 µWh cm^−2^, 0.1 mW cm^−2^),^[^ ^43^ ^]^ Activated graphene (2 µWh cm^−2^, 0.2 mW cm^−2^ at 150 °C),^[^ ^44^ ^]^ and CNT/PANNNFs (8.5 µWh cm^−2^, 0.1 mW cm^−2^ at 100 °C).^[^ ^45^ ^]^ All results indicate that porous GaN‐based heterostructure is a prospective material for energy storage in high operating temperature environments.

**Figure 5:**
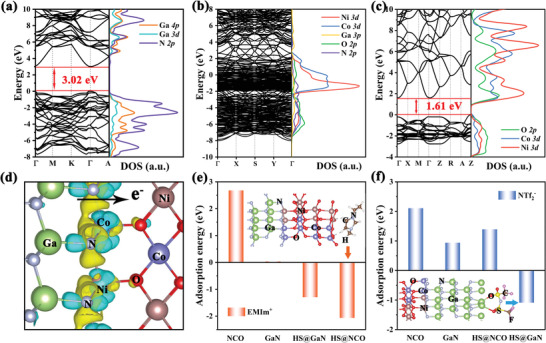
Density functional theory. The band gap and PDOS of the GaN (a), GaN/NCO heterostructure (b), and NCO (c); d) the charge density difference of the GaN/NCO heterostructure; e,f) the calculated adsorption energy of ions on the GaN, GaN/NCO heterostructure, NCO.

To further appreciate the role of the GaN/NCO heterostructure‐based device with ILs as an electrolyte in energy storage, the density functional theory is carried out. Taking into account the differences in the types of the atoms bound at the heterogeneous interface, two configurations of the GaN/NCO heterostructure and the binding energy of the possible bonded atoms are constructed and analyzed (Figure S23, Supporting Information). One type of heterojunction structure is Ga—O bonding at the GaN/NCO interface, where metal atoms presented on the surface of GaN crystal spontaneously attract O atoms in the that of NCO. Another heterostructure is the transition metal atoms (Ni and Co) that transfer electrons and combine with N atoms of the GaN to form the M—N bond, which behaves the lower binding energy and indicates the heterostructure is more stable in comparison with the Ga—O bond.^[^ ^46^ ^]^ The density of states (DOS) and bandgaps of all samples (GaN, GaN/NCO heterostructure, NCO) are also calculated to illustrate the effects of the heterostructures on electronic properties (**Figure** **5**a–c). In the partial DOS (PDOS) electronic state of the GaN/NCO heterostructure, the hybridization of Ni *3d*, Co *3d*, and N *2p* orbitals occurs near the Fermi level (*E*_f_) in the heterostructure, which represents the bonding GaN to NCO.^[^ ^47^ ^]^ Furthermore, the GaN/NCO heterostructure exhibits typical metallic characteristics as the *E*_f_ spans a continuous gap state, while the GaN with a bandgap of 3.02 eV and the NCO of 1.61 eV, which both stand for the semiconductor properties.^[^ ^48^ ^]^ This result confirms the appearance of interfacial coupling between GaN and NCO, and the electrons migration of the GaN/NCO is no longer restricted to particular parts, which promotes the electron transfer kinetics and improves the electrical conductivity.^[^ ^49^ ^]^

The charge redistribution of the interfacial can be significantly observed from the charge density difference. In Figure 5d, the charge depletion and accumulation around the heterogeneous interfaces indicate that the N *2p* orbital in GaN crystal interacts with Ni *3d* and Co *3d* of the NCO and accompany the surrounding electron density changes. The Bader charge is conducted to analyze the exact charge transfer (Table S4, Supporting Information). According to the average Bader charge, it can be seen that the electron transfer from GaN to NCO, which giving rise to the intense interface contact between the GaN and NCO. As a result, the polarization intensity of the GaN is improved through the coupling with NCO and forms the local electric fields with a large number of active sites to adsorb electrolyte ions, which account for enhanced energy storage. The comprehensive investigation of the ILs adsorption on the GaN/NCO heterostructure of four different position points, where including the GaN surface, the NCO rim of heterostructure (HS@NCO), the GaN side of heterostructure (HS@GaN), and the NCO surface. The energy values of adsorption on different sites of GaN/NCO heterostructure toward ILs are summarized in Figure 5e,f. In particular, it is confirmed that the adsorption of the positively charged (EMIm^+^) is strongest at the HS@NCO that the adsorbed energy is as low as −2.07 eV, and that the adsorption of the negatively charged (NTf_2_^−^) is favorable at the HS@GaN with the adsorption energy is the −1.29 eV. And the corresponding stable adsorption structure is shown. These conclusions suggest that the establishment of the phase junction region facilitates higher adsorption enthalpy of ILs to achieve excellent electrochemical performance.^[^ ^50^ ^]^

To further verify the difference in samples' adsorption properties, the ILs are dripped on GaN, GaN/NCO‐2 heterostructure, NCO at the high temperature of 130 °C. The GaN/NCO‐2 heterostructure clarifies a reduced contact angle of the ILs than bare porous GaN and NCO (Figure S24, Supporting Information). This phenomenon reflects the superior ILs wettability of the GaN/NCO‐2 heterostructure. The above conclusion is consistent with the theoretical calculation and experimental results. The GaN/NCO heterostructure is regarded as a promising material for improving energy storage in high‐temperature conditions.

## 3 Conclusion

In summary, the GaN/NCO heterostructure is generated via a simple electrochemistry etching technique and in situ growth strategy. Benefiting from the synergy of the 3D GaN conductive scaffold and the ideal theoretical capacity of NCO, the GaN/NCO heterostructure‐based SCs with ILs as electrolytes acquire satisfactory electrochemical performance at 130 °C. The maximum power density of 44.0 mW cm^−2^ is achieved and 83.6% of the initial capacitance is maintained after 10 000 cycles. Moreover, theoretical calculations are combined to demonstrate the effectiveness of the built‐in electric field, which promotes the adsorption of electrolyte and reduce the energy barrier of ions/electronics transport. Undoubtedly, the design strategy of the heterostructure opens new avenues for high‐temperature GaN‐based energy storage devices with superior performance.

## Conflict of Interest

The authors declare no conflict of interest.

## Supporting information

Supporting Information

## Data Availability

The data that support the findings of this study are available from the corresponding author upon reasonable request.
