# Supplementary material for: Gallium Nitride Based Electrode for High‐Temperature Supercapacitors
Source: Adv Sci (Weinh). 2023 Mar 25;10(15):2300780. doi: 10.1002/advs.202300780 (PMC10214239; doi:10.1002/advs.202300780)
Supplement: Supplementary file 1 — Supporting Information [file ADVS-10-2300780-s001.pdf]

ADVANCED SCIENCE
Open Access

Supporting Information

for *Adv. Sci.*, DOI 10.1002/advs.202300780

Gallium Nitride Based Electrode for High-Temperature Supercapacitors

Songyang Lv, Shouzhi Wang, Lili Li, Shoutian Xie, Jiaoxian Yu, Yueyao Zhong*, Guodong Wang, Chang Liang, Xiangang Xu and Lei Zhang**

Supporting Information

Gallium Nitride Based Electrode for High Temperature Supercapacitors

Songyang Lv[a], Shouzhi Wang[,a, b], Lili Li[c], Shoutian Xie[d], Jiaoxian Yu[e], Yueyao Zhong[*,f], Guodong Wang[a], Chang Liang[a], Xiangang Xu[a], Lei Zhang[*, a, b]*

[a]Institute of Novel Semiconductors, State Key Lab of Crystal Materials, Shandong University, Jinan, 250100, P. R. China.

[b]Shenzhen Research Institute, Shandong University, Shenzhen, 518000, P. R. China

[c]Institute of Crystal Materials, State Key Lab of Crystal Materials, Shandong University, Jinan, 250100, P. R. China.

[d]School of Public Administration, Shandong Normal University, Jinan, 250100, P. R. China.

[e]Key Laboratory of Processing and Testing Technology of Glass & Functional Ceramics of Shandong Province, School of Materials Science and Engineering, Qilu University of Technology (Shandong Academy of Sciences), Jinan 250353, P. R. China.

[f]School of Materials Science and Engineering, Shandong Jianzhu University, Jinan, 250100, P. R. China

E-mail: wangsz@sdu.edu.cn; 13922@sdjzu.edu.cn; leizhang528@sdu.edu.cn

This file includes:

S1. Materials

S2. Theoretical calculation

S3. Characterization Methods

S4. Electrochemical Measurements.

S5. Calculation of Capacitance Contribution

S6. Supplementary Figures S1–S24

S7. Table S1–S4

S1. Materials

Reagents were purchased from Sinopharm Chemical Reagent Co., Ltd. (Shanghai). All the reagents were used as received.

S2. Experimental Section

S2.1 Preparation of porous GaN single crystal:

GaN crystal was grown by metal organic chemical vapor deposition. Porous GaN membrane single crystal was fabricated by a classical electrochemical etching processing. Specifically, two electrode system that the 0.3 M oxalic acid solution as electrolyte was applied. The GaN crystal and platinum wire as anode and cathode, respectively. The etching process was controlled by voltage parameter. Firstly, the pulse voltage is kept constant at 5 V with 10 min, and then the voltage is converted to 15 V with 3 min.

S2.2 Preparation of the GaN/NCO heterostructure:

The NiCo precursor in situ growth on the surface of porous GaN membrane by a strep hydrothermal method. A series of porous GaN based heterostructures were prepared by using the molar ratios of porous GaN to nickel/cobalt chloride hexahydrate were 1: 0: 0, 1: 1: 1, 1: 3: 3, 1: 6: 6 and 0: 1: 1, they are labeled as GaN, GaN/NCO-1, GaN/NCO-2, GaN/NCO-3 and NCO, respectively. For the GaN/NCO-2, cobalt chloride hexahydrate (0.065 g), nickel chloride hexahydrate (0.065 g) and urea (0.1 g) were dissolved in 40 mL deionized water during continuous stirring. Afterward, carefully transfer the homogeneous solution and a piece of porous GaN membrane (approximately 7 mg) to 50 ml autoclave for heating 130 ℃ with 6 hours. The

GaN/NiCo-precursor was washed and dried at 80 ℃ in a vacuum oven. Finally, the GaN/NiCo-precursor sample was put into quartz boat and annealed in argon atmosphere at 350 ℃ for 2 hours with the heating rate of 5 ℃/min to obtain the porous GaN/NCO product.

S2.3 Fabrication of the porous GaN based heterostructure electrode and devices with ionic liquid as electrolyte:

The active materials (GaN/NCO heterostructure), PVDF binders, acetylene blacks with a weight ratio of 80: 10 :10 were mixed in N-methylpyrrolianone (NMP) to prepared the electrode. Then the evenly slurry was spreaded on the current collector (stainless steel cloth with 1 × 1 cm) that was cleaned and completely dried for 10 hours in a vacuum. The mass loading of the electrode was approximately 1 mg cm^{-2}. GaN based heterostructure SCs were assembled in an argon filled glove box (< 0.01 ppm of oxygen and water). The porous GaN/NCO heterostructure served as anode and cathode respectively and the 1-ethyl 1-3 methimidazole (trifluoromethyl sulfonyl) imide ($EMImNTf_2$) used as electrolyte.

S2.4 Theoretical calculation method:

The present calculations were performed based on the Vienna ab initio Simulation Package (VASP)[1, 2] implementation of density functional theory (DFT) in conjunction with the projector augmented wave (PAW). Consequently, the Ni $3d^8 4s^2$, Co $3d^7 4s^2$, Ga $4s^2 3d^{10} 4p^1$, H $1s^1$, C $2s^2 2p^2$, N $2s^2 2p^3$, O $2s^2 2p^4$, F $2s^2 2p^5$, and S $3s^2 3p^4$ states were treated as valence electrons. To optimize the configurations, this study adopted the electron exchange and correlation within the generalized gradient

approximation (GGA) of the Perdew–Burke–Ernzerhof (PBE) functional.[3] The electronic wave functions are expanded in plane waves using energy cut off of 500 eV. The convergence criteria of force and energy for the structural relaxation were set at 0.01 eV/Å and 10-5 eV. To obtain the accurate electronic structure, All the electronic properties of GaN, NCO, and GaN/NCO are determined using the screened Heyde-Scuseriae-Ernzerhof (HSE) hybrid functional.[4, 5] Monkhorst-Pack k-points [6] were sampled using a 2×2×2 for electronic calculations and 4×4×1 for the relaxation and total energy. The adsorption energy (E_{ad}) for species X is defined as follows: $E_{ad} = E(\text{surface/X}) - E(\text{surface}) - E(\text{X})$, where E(surface/X) is the total energy of the fully relaxed surface/adsorbate system, E(surface) is the total energy of the relaxed substrate slab, and E(X) is the total energy of a free adsorbate species. Negative adsorption energy means effective adsorbate binding**.**

S3. Characterization Methods

The morphology of the as-collected samples was characterized by investigated by SEM (Hitachi S-4800), TEM (Philips Tecnai 20U-Twin), HRTEM (JEM 2100F). The crystal structures were obtained by XRD (Bruker diffractometer D8 Advance, Ni Kα radiation, λ = 1.5418 Å), XPS (Thermo ESCALAB 250 Al Kα radiation, 1486.8 eV), BET (ASAP 2020 sorptometer), PSD (BJH desorption d*V*/dlog(*r*) plot volume), Raman (Horiba Jobin Yvon with Ar^+ laser 532 nm), EPR (Bruker, A300-10/12), TGA (Hitachi TG/DTA7200), FTIR (Thermo Scientific Nicolet iS20)

S4. Electrochemical Measurements.

Electrochemical performance tests were performed with the CHI 660E electrochemical workstation. For the three-electrode test system with GaN/NCO heterostructures as the working electrode (The area of active material on the stainless-steel cloth was 1.5 cm^2, and the mass loading was about 1 mg cm^{-2}) with respect to saturated calomel as reference electrode, metal platinum plate as counter electrode in 6 M KOH aqueous solution. The CV and GCD curves were carried out in –0.4 V to 0.4 V under 25 °C. In two-electrode measurements, the CV and GCD test data was collected at 0–2.2 V range from 25 °C to 130 °C. The alternating current (AC) EIS spectrum was acquired range from 10^{-2} Hz to 10^5 Hz at an open circuit voltage with an AC amplitude of 0.005 V. Mott-Schottky (M-S) for the measured frequency of 973Hz.

The area specific capacity of the electrodes in three-electrode test system, is derived from the GCD curve, calculated according to equation 1.

$$C = \frac{I\Delta t}{S\Delta V}$$

Where C is the specific capacitance (F cm^{-2}) base on the area of the electrode materials, I is the discharge current (A), Δt is the discharge time (s), S is the area (cm^2) of the active materials of the single electrode, ΔV is the working voltage window (V).

The area specific capacitance for GaN/NCO based SC is calculated from equation 2

$$C = \frac{I\Delta t}{S\Delta V}$$

Where C is the area specific capacitance (F cm^{-2}) of the device, I is the discharge current (A), Δt is the discharge time (s), S is the total area (cm^2) of active material on the SC, ΔV is the working voltage window (V).

The energy density of the SCs are calculated by equation 3

$$E = \frac{1}{2} C \Delta V^2$$

Where E is the energy density (mWh cm^{-2}) of the device, C is the area specific capacitance (mF cm^{-2}) of the device, ΔV is the working voltage window (V).

The power density of the SCs are calculated by equation 4

$$P = \frac{E}{\Delta \mathrm{t}}$$

Where P is the power density (mW cm^{-2}) of the device, E is the energy density (mWh cm^{-2}), Δt is the discharge time (s).

According to the M-S theory, the calculated electron concentration, was derived from equation 5

$$\frac{1}{\mathrm{C_s^2}} = \frac{2}{(\varepsilon \varepsilon_0 e_0 N_d)} (U - U_{fb} - \frac{\kappa_B T}{e_0})$$

Where C_s is the space charge capacitance per unit area (F cm^{-2}), N_d is the electron concentration (cm^{-3}), ε is the dielectric constant, ε_0 is the permittivity of vacuum (F m^{-1}), e_0 is the electron charge (C), U is the applied potential (V), U_{fb} is the flat band potential (V), T is the temperature (K), and κ_B is the Boltzmann constant (J K^{-1}).

S5. Calculation of Capacitance Contribution

The b value from $i=av^b$ can be obtained by plotting current and sweep rate in logarithm (Equation 6), namely, the gradient of linear plots.

$$\log i = b \log v + \log a$$

In order to determine the $k1$ value, Equation 7 can be reformulated as:

$$i(V) = k_1 v + k_2 v^{\frac{1}{2}}$$

S6. Supplementary Figures S1-S23

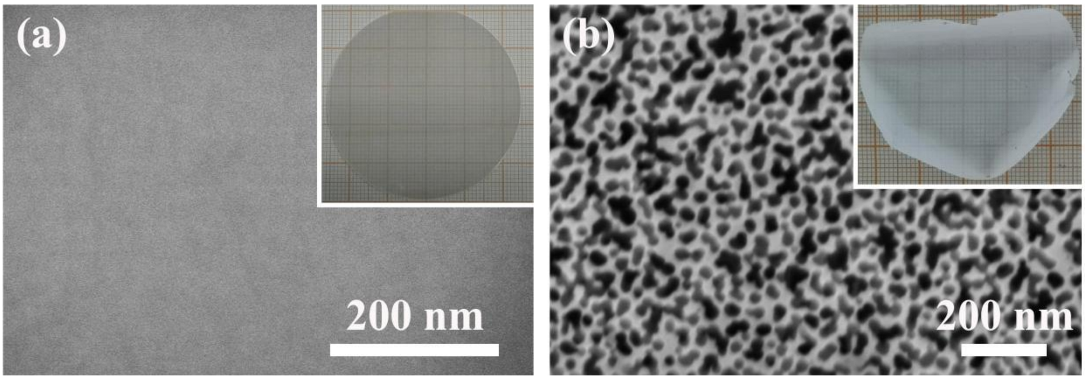

Figure S1. The image of the GaN crystal that before electrochemical etching (a) and porous GaN membrane after electrochemical etching (b).

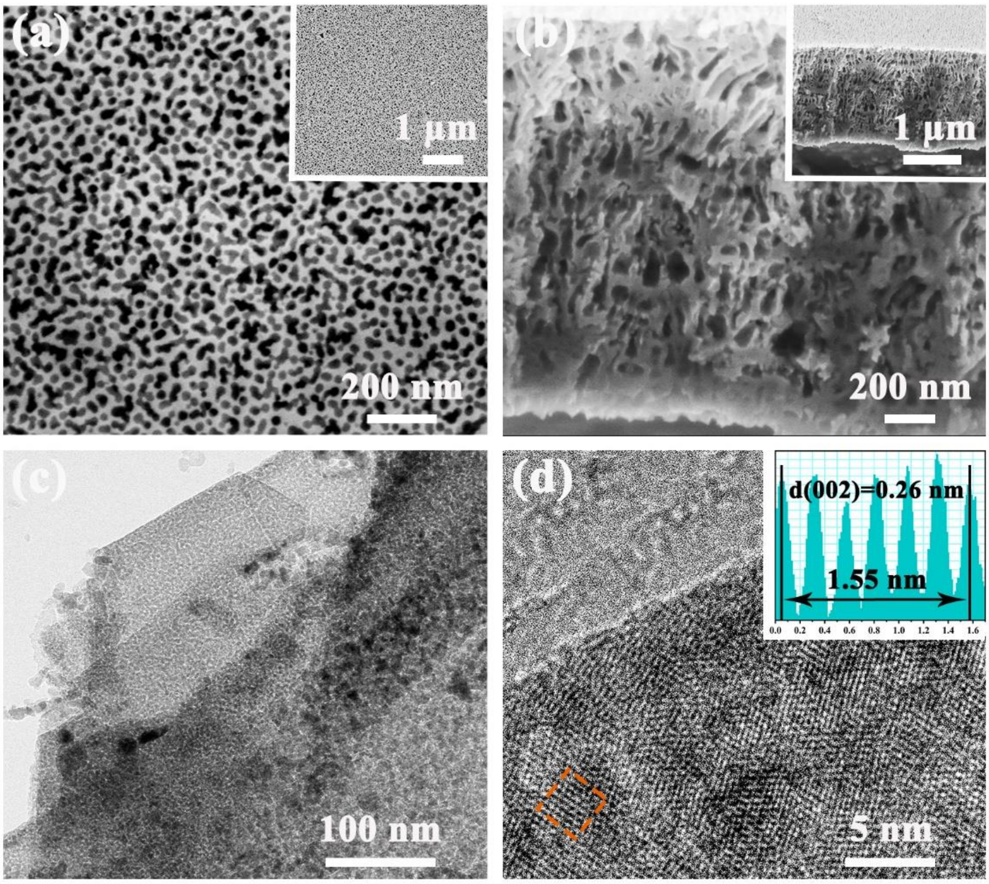

Figure S2. The morphological characterization of porous GaN membrane. (a) the SEM image and the corresponding cross sectional view (b); (c) the TEM; (d) the HRTEM image and the relevant lattice fringe spacing inset in the (d).

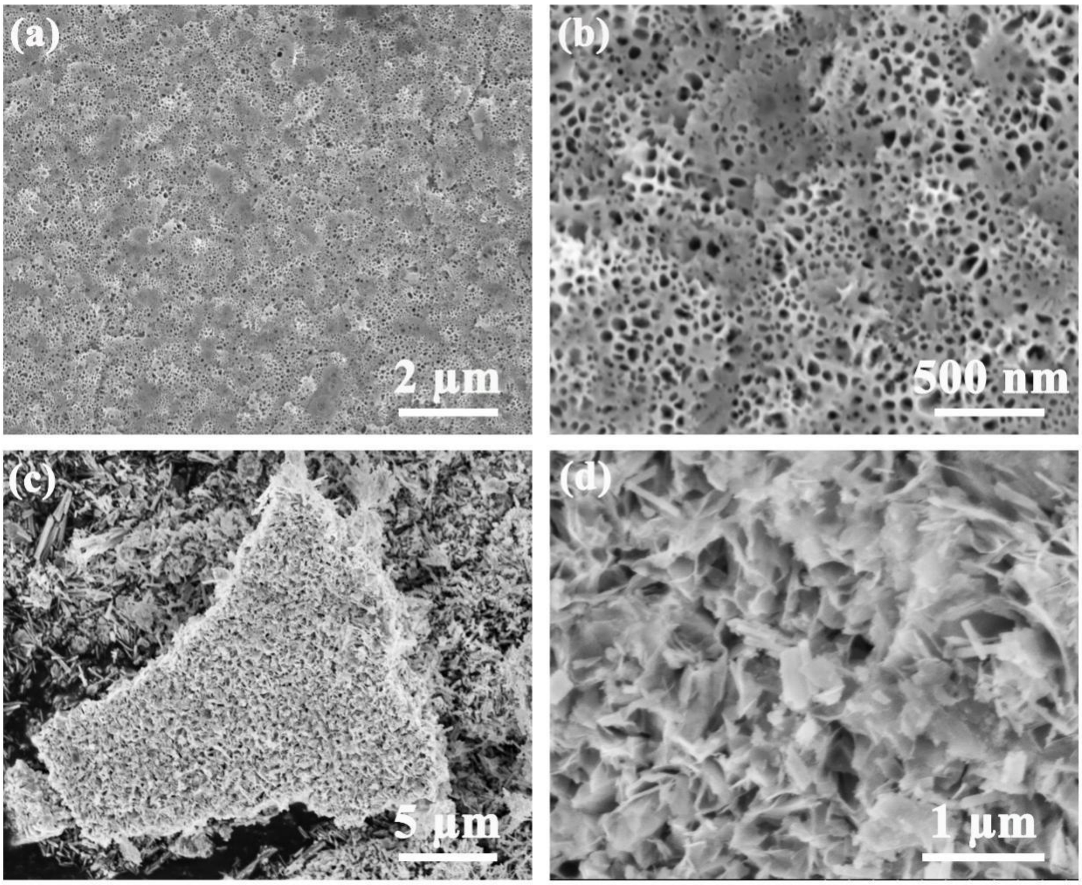

Figure S3. The SEM images of the GaN/NCO-1 (a, b) and GaN/NCO-3 (c, d).

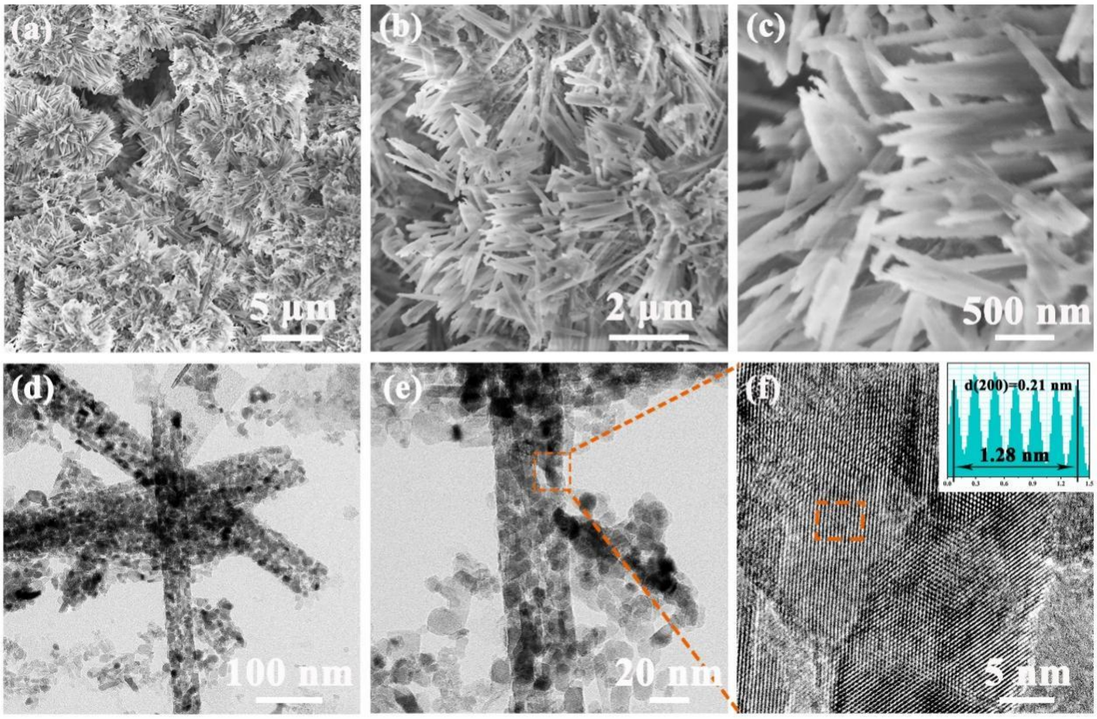

Figure S4. The morphological characterization of NCO nanowires. (a-c) the SEM images; (d) the TEM image; (e, f) the HRTEM images and the relevant linear profiles defined lattice fringe spacing inset in the (f)

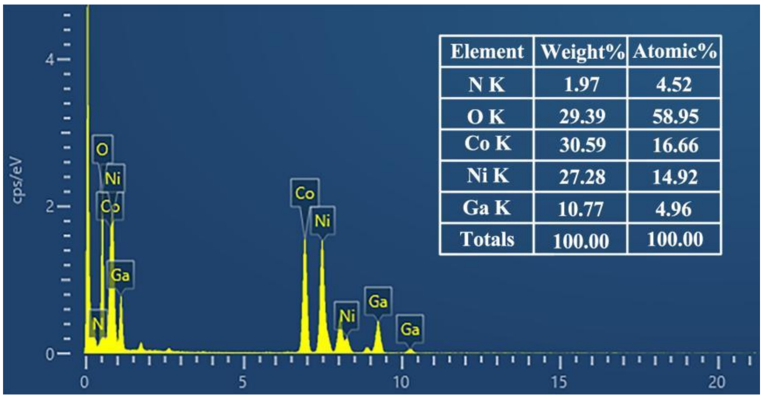

Element	Weight%	Atomic%
N K	1.97	4.52
O K	29.39	58.95
Co K	30.59	16.66
Ni K	27.28	14.92
Ga K	10.77	4.96
Totals	100.00	100.00

Figure S5: The EDS result of the GaN/NCO-2 heterostructure.

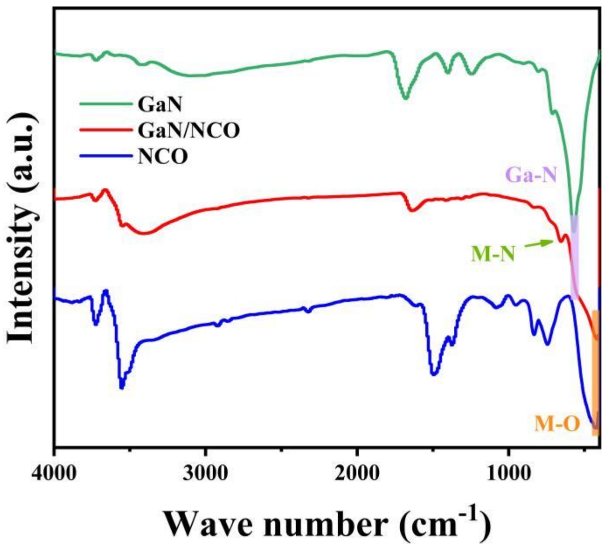

Figure S6. The FITR spectra.

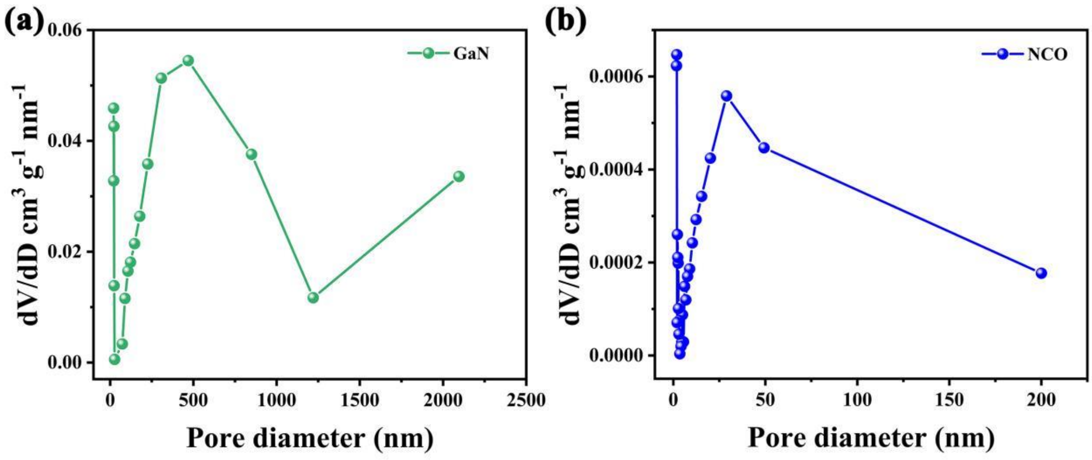

Figure S7. The pore size distribution plots of porous GaN (a) and NCO (b).

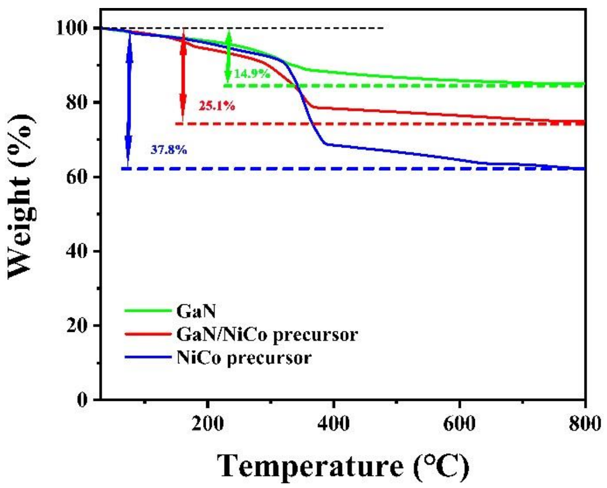

Figure S8. The TGA profile.

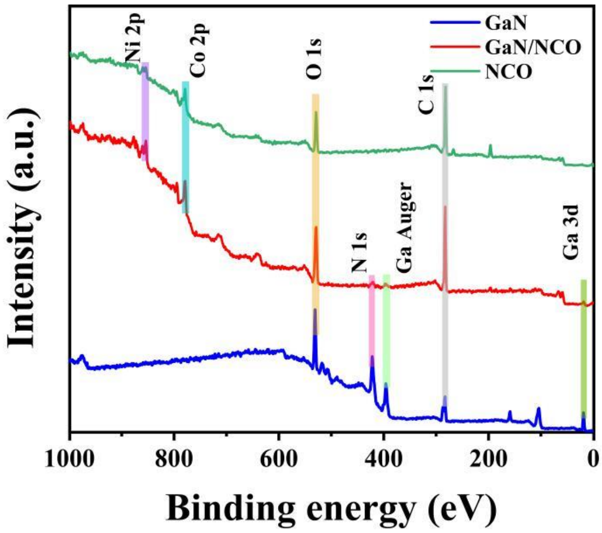

Figure S9. The XPS survey spectra.

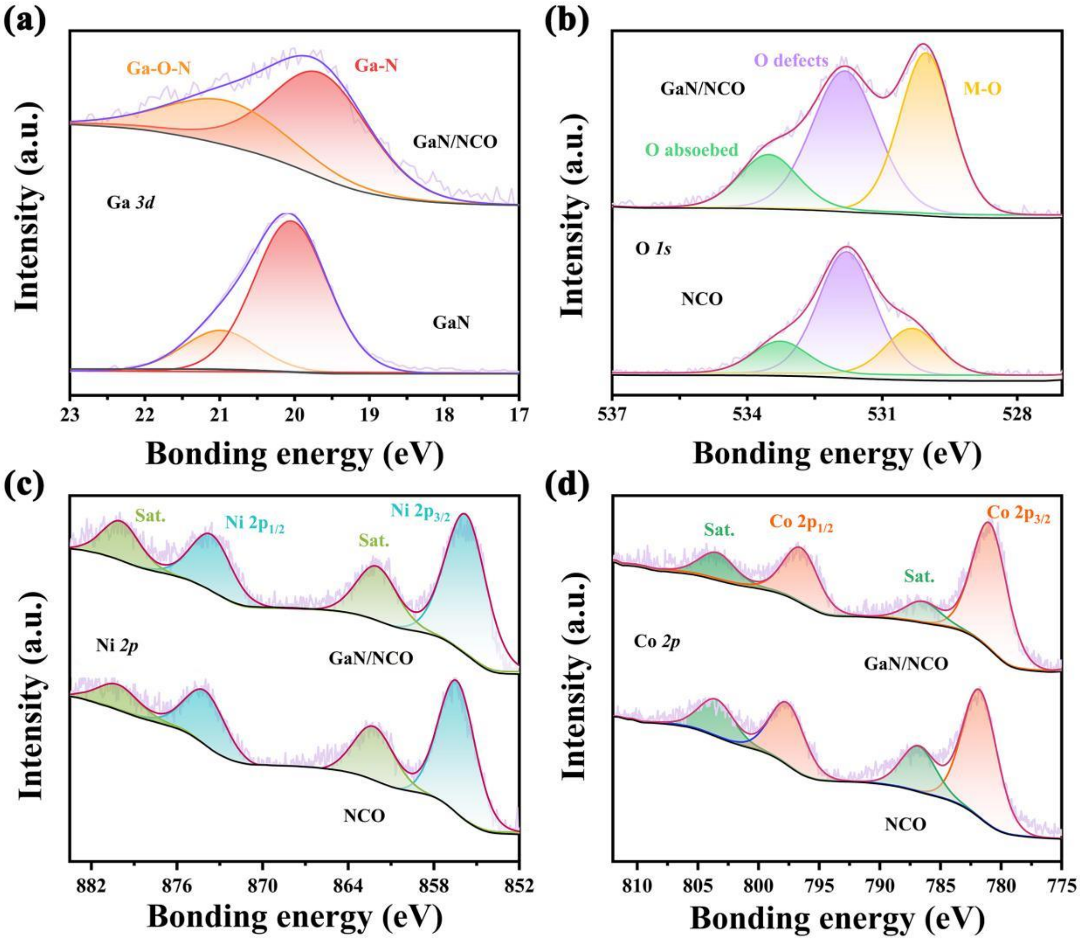

Figure S10. The High-resolution XPS spectra of Ga *3d* (a); O *1s* (b); Ni *2p* (c); Co *2p* (d).

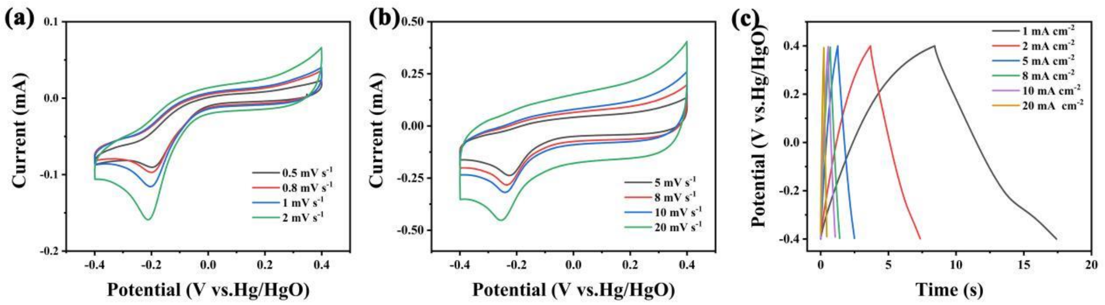

Figure S11. Three-electrode electrochemical performance of GaN membrane. (a, b) the CV curves; (c) the GCD curves.

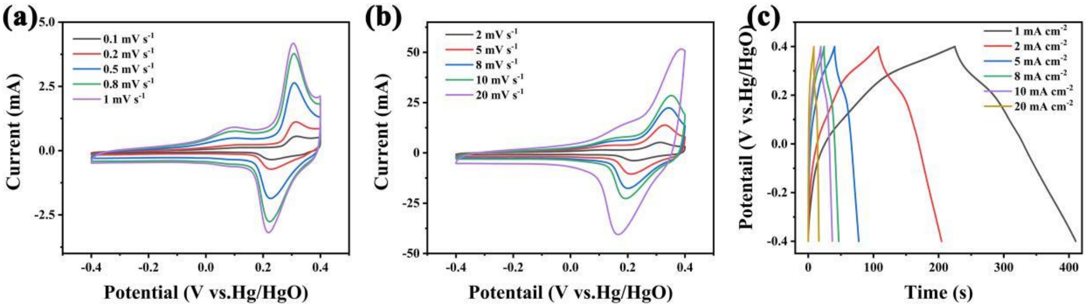

Figure S12. Three-electrode electrochemical performance of GaN/NCO-1. CV curves (a, b); (c) the GCD curves.

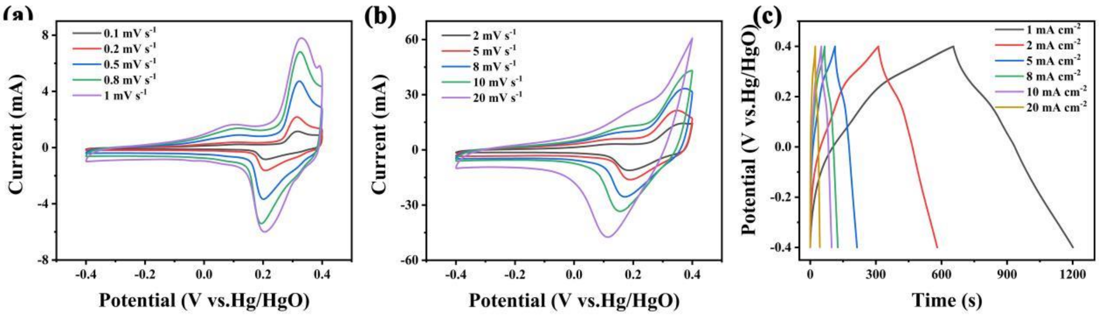

Figure S13. Three-electrode electrochemical performance of GaN/NCO-2. CV curves (a, b); (c) the GCD curves.

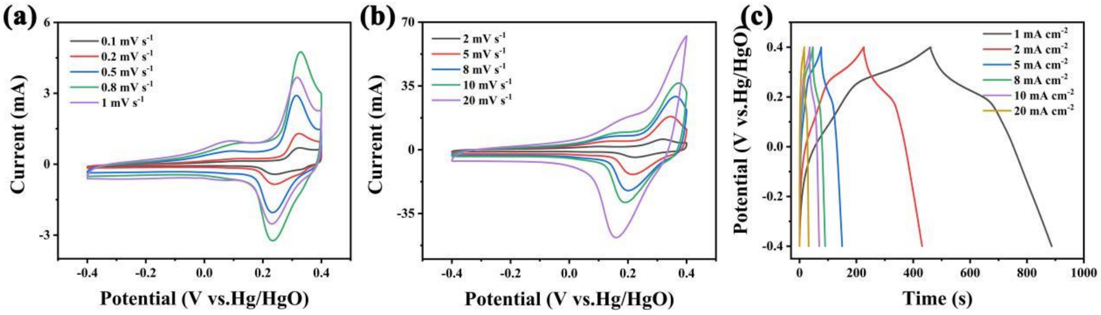

Figure S14. Three-electrode electrochemical performance of GaN/NCO-3. CV curves (a, b); (c) the GCD curves.

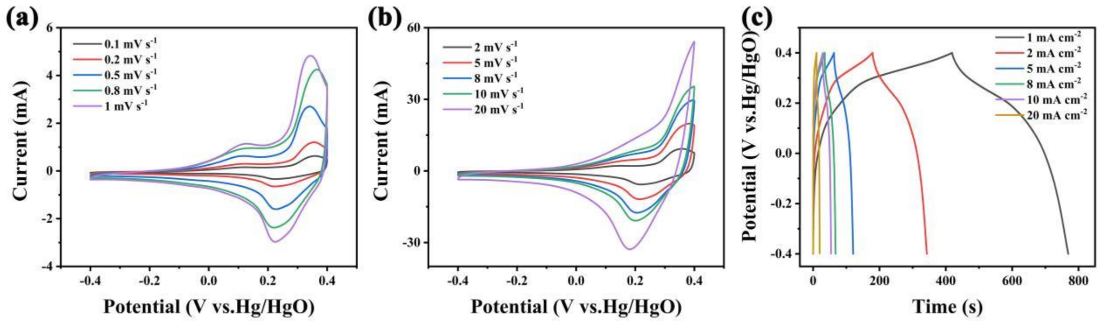

Figure S15. Three-electrode electrochemical performance of NCO. CV curves (a, b); (c) the GCD curves.

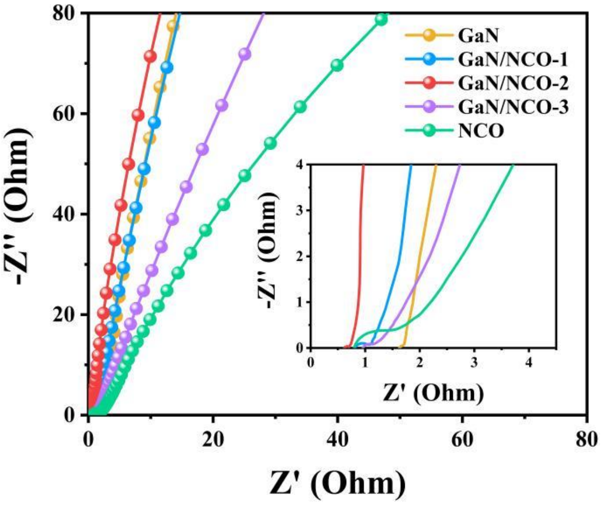

Figure S16. Three-electrode EIS tests. Nyquist plots of GaN, GaN/NCO-1, GaN/NCO-2, GaN/NCO-3, NCO.

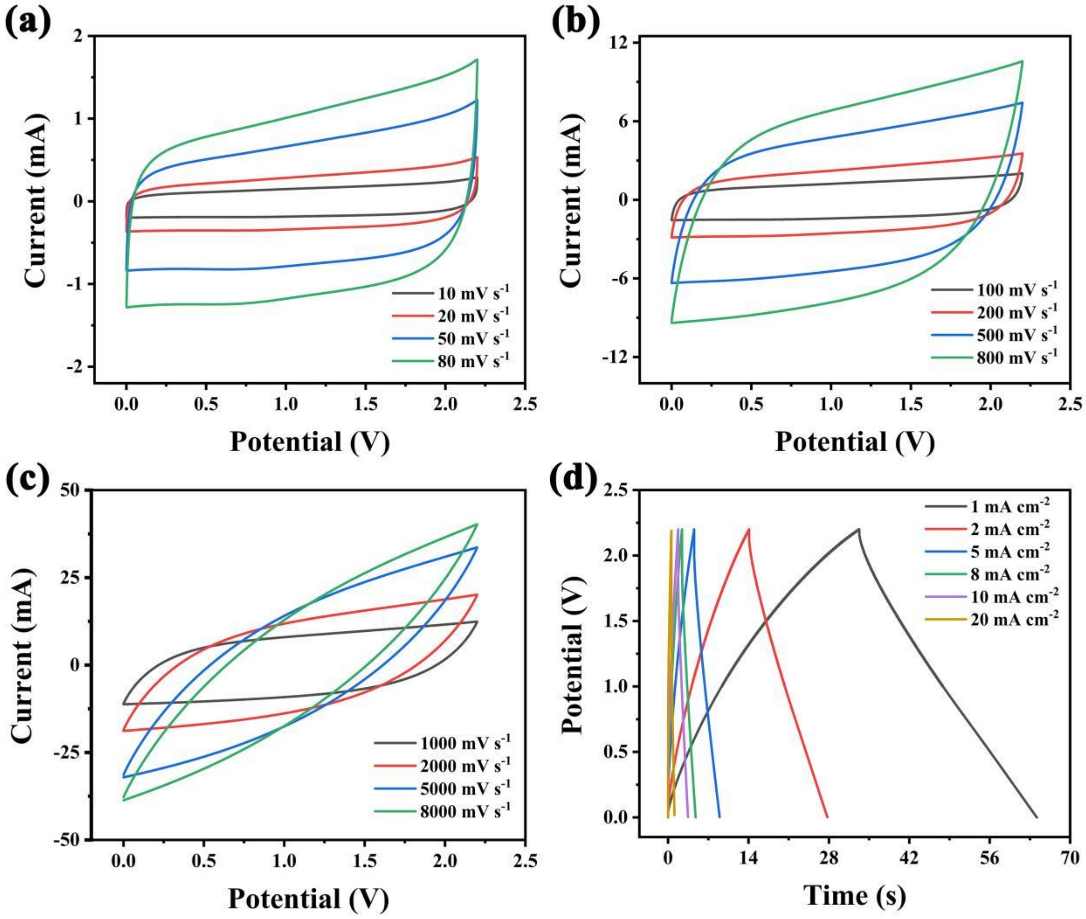

Figure S17. The electrochemical performance of GaN/NCO heterostructure SCs at 25 ℃. The CV profiles (a-c); (d) the corresponding GCD curves.

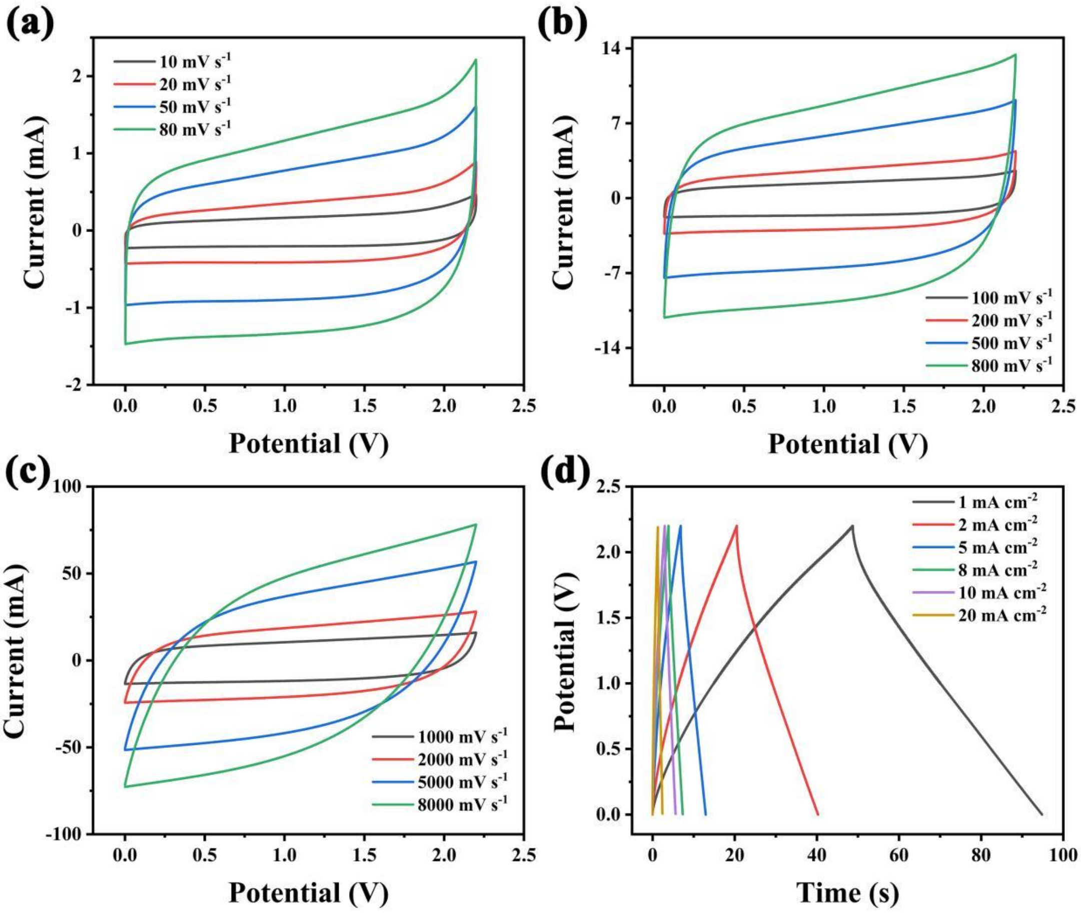

Figure S18. The electrochemical performance of GaN/NCO heterostructure SCs at 60 ℃. The CV profiles of the device (a-c); (d) the corresponding GCD curves.

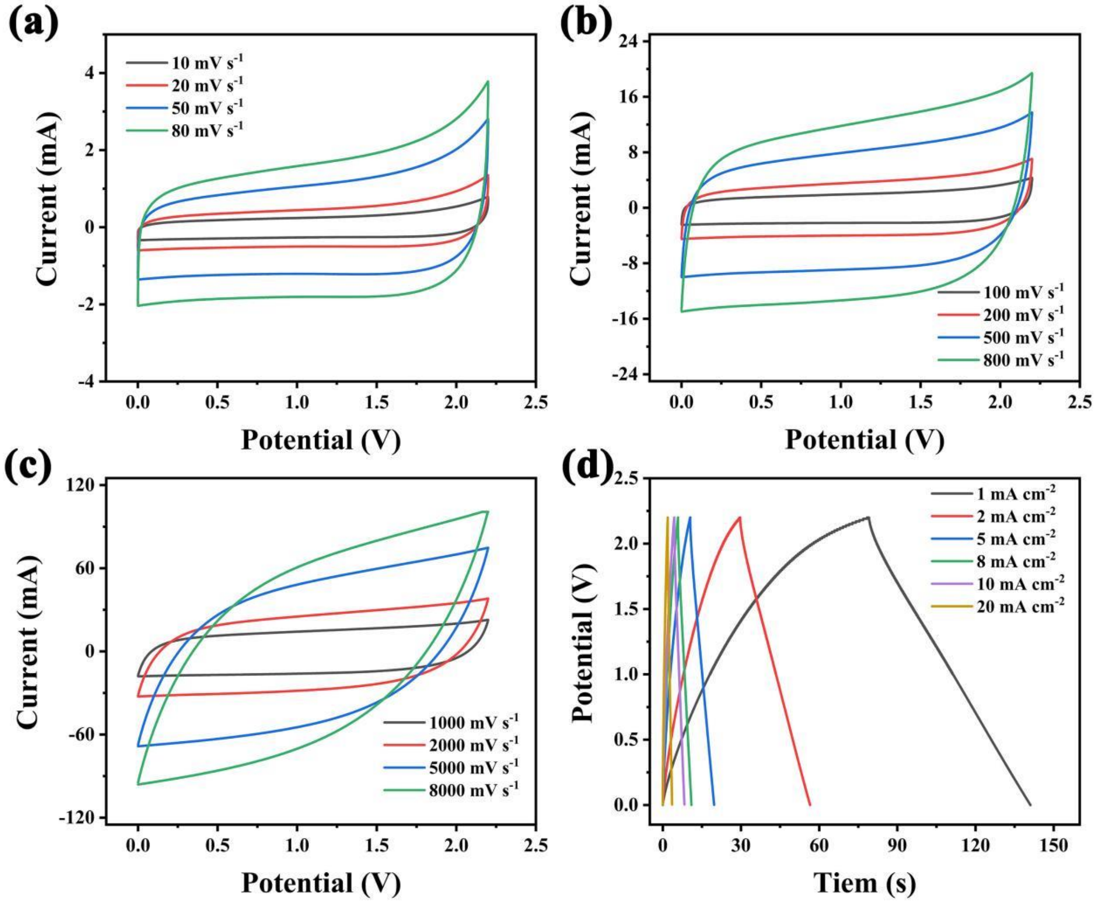

Figure S19. The electrochemical performance of GaN/NCO heterostructure SCs at 100 ℃. The CV profiles of the device (a-c); (d) the corresponding GCD curves.

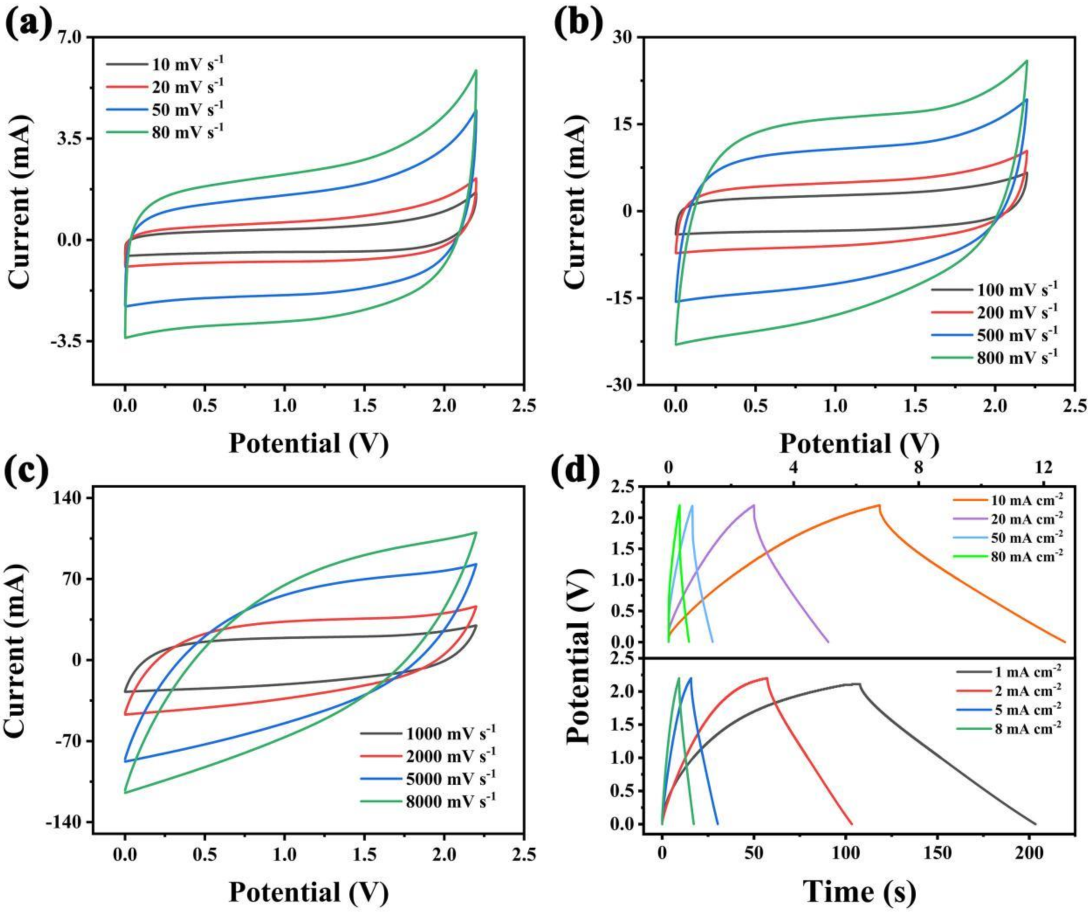

Figure S20. The electrochemical performance of GaN/NCO heterostructure SCs at 130 ℃. The CV profiles of the device (a-c); (d) the corresponding GCD curves.

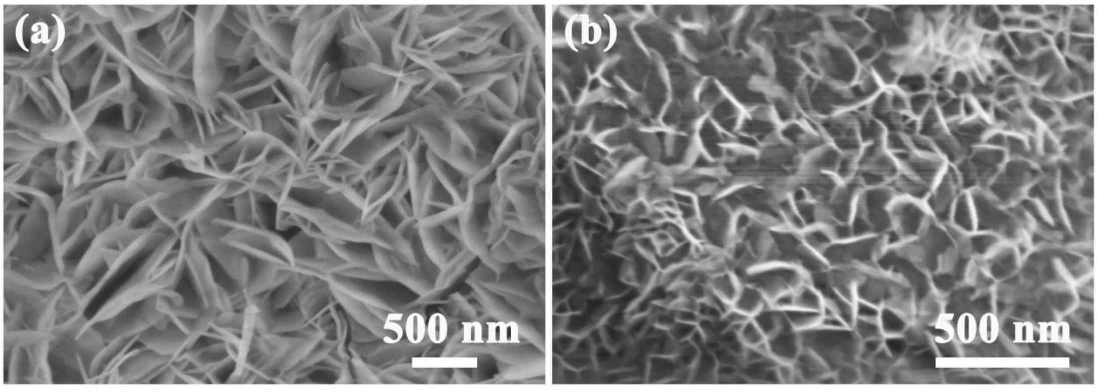

Figure S21. The SEM images of the GaN/NCO-2 heterostructure based SCs of 130 ℃ before (a) and after (b) 10,000 cycles at 10 mA cm^{-2}.

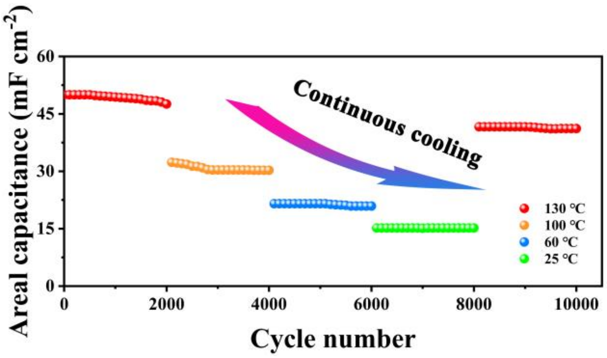

Figure S22. The cycling stabilities with progressive variation in temperatures between 25 °C and 130 °C.

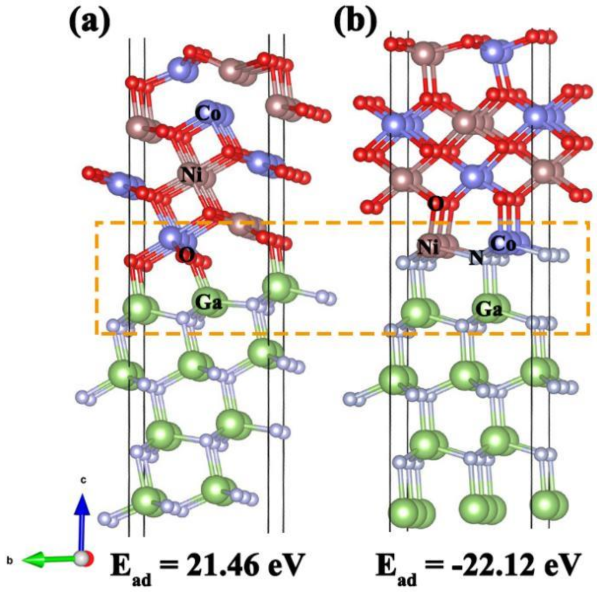

Figure S23. Optimized configuration of GaN/NCO heterostructure. (a) Heterostructure of Ga-O interface; (b) heterostructure of M-N interface.

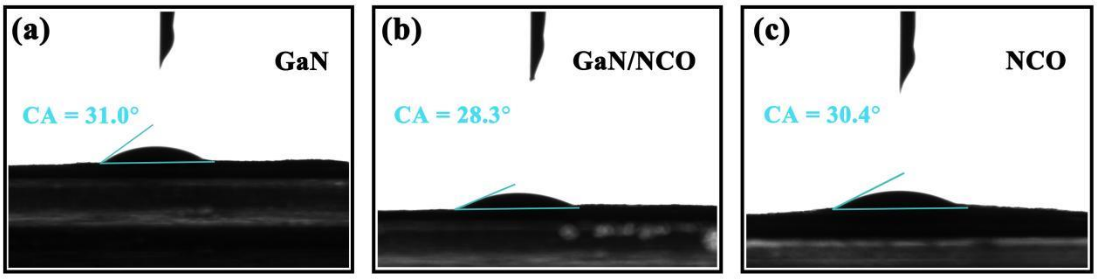

Figure S24. The dynamic contact angles of ILs on the surface bare porous GaN (a), GaN/NCO-2 heterostructure, (c) NCO at 130 °C.

Table S1: Comparison of electrochemical performance of GaN/NCO heterostructure with others relevant reported in the literatures.

Material	Specific capacitance	Stability	Rate performance	Ref.
GaN/NCO	683 mF cm^{-2} at 1 mA cm^{-2}	82%-10,000	76% (from 1 to 20 mA cm^{-2})	This work
SiC@C	78.98 mF cm^{-2} at 0.2 mA cm^{-2}	/	44% (from 0.2 to 10 mA cm^{-2})	*Energy Stor. Mater*, **2020**, 27, 261
MoS_2/Ti_3C_2	347 mF cm^{-2} at 2 mA cm^{-2}	/	41.7% (from 2 to 30 mA cm^{-2})	*Adv. Funct. Mater.* **2020**, *30*, 1910302
SiC/MXene	307 F g^{-1} at 1 A g^{-1}	/	69.1% (from 1 to 10 mA cm^{-2})	*Chem. Eng. J*, **2022**, 428, 131114
SiC@Fe_2O_3	721 F g^{-1} at 2A g^{-1}	/	51% (from 2 to 12A g^{-1})	*Adv. Energy Mater.*, **2018**, *8*, 1702787
SiC/G-HNF	0.41 mF cm^{-2} at 5 μA cm^{-2}	86% - 5,000	~50% (from 5 to 20 μA cm^{-2})	*Small* **2018,** *14*, 1801857
$Ni_2P/NiSe_2$	3500 mF cm^{-2} at 1 mA cm^{-2}	51.9%-5,000	45% (from 1 to 20 mA cm^{-2})	*Adv. Funct. Mater.*, **2022**, *32*, 2204833
SiC@PEDOT	13 mF cm^{-2} at 0.2 mA cm^{-2}	/	50% (from 0.2 to 2 mA cm^{-2})	*J. Energy Chem.*, **2022**, *66*, 30
Co_9S_8@MnO_2	2.331 F cm^{-2} at 1 mA cm^{-2}	66.7%-5,000	20% (from 1 to 20 mA cm^{-2})	*Chem. Eng. J*, **2022**, *437*, 135494
C/PSiC	325 mF cm^{-2} at 1mA cm^{-2}	83%-5,000		*Nano Lett.* **2014,** *14*, 1843
SiC/N doped C	158 F g^{-1} at 0.5 A g^{-1}	/	50% (from 0.5 to 20 A g^{-1})	*Chem. Eng. J*, **2022**, *433*, 133738
SiC/CNT	8.43 F g^{-1} at 0.02 A g^{-1}	/	70% (from 0.02 to 0.32 A g^{-1})	*J. Mater. Chem. A*, **2022**, *10*, 15708
GaN@ RGO-GaN	450 F g^{-1} at 0.01 V s^{-1}	75%-1,000	66% (from 0.01 to 0.05 V s^{-1})	*J Phys Chem Sollids*, **2020**, *141*, 109406
GaN/GP	225 mF cm^{-2} at 1 mA cm^{-2}	/	66% (from 1 to 20 mA cm^{-2})	*Small*, **2017**, *13*, 1603330
SiC-CDC	260 F g^{-1} at 1 A g^{-1}	97%-5,000	36.5% (from 1 to 10 A g^{-1})	*J. Mater. Chem. A*, **2018**, *6*, 12724
3C/2H-SiC	0.73 mF cm^{-2} at 10 mV s^{-1}	91.2%-20,000	53.2% (from 10 to 50mV s^{-1})	*J. Mater Sci Technol*, **2022**, *110*, 178
TiON/MCO	515 F g^{-1} at 1 A g^{-1}	78%-5,000	50% (from 1 to 10 A g^{-1})	*ACS Appl. Mater. Interfaces*, **2020,** *12*, 54524
Co_3O_4@ CNT@D	729.6 F g^{-1} at 4 A g^{-1}	71.5%-5,000	55.6% (from 1 to 20 A g^{-1})	*J Energy Storage*, **2022**, *53*, 105094

Table S2. Fitting report of Figure 4e insert based on a R(C(RW))C equivalent circuit.

Index	Fixed	Parameter	Start	End	Rel. Std. Error (%)
1	0	R	1.636	1.636	2.497
2	0	C	0.0000001075	0.00001075	5.318
3	0	R	2.295	2.294	2.296
4	0	W	0.09981	0.09980	4.601
5	0	C	0.02415	0.02415	2.092

Table S3. Comparison of electrochemical performance of GaN/NCO heterostructure based SC with others high temperature SC electrodes reported in the literatures.

Material	Temperature (°C)	Max Scan rate	Specific capacitance	Energy density	Power density	Capacitance retain-cycle	Ref.
**GaN/NCO**	**130**	**8 V s^{-1}**	**90.6 mF cm^{-2} at 1 mA cm^{-2}**	**15.2 µWh cm^{-2}**	**44 mW cm^{-2}**	**83.6% -10,000**	**This work**
CNT-MnO_2	100	100 mV s^{-1}	43 mF cm^{-2} at 5 mV s^{-1}	24 µWh cm^{-2}	/	81%-20,000	*Adv. Funct. Mater.* **2022,** *32,* 2203270
MWCNT/RGO	80	200 mV s^{-1}	30.5 mF cm^{-2} at 0.25 mA cm^{-2}	0.2 µWh cm^{-2}	0.3 mW cm^{-2}	87%-5,000	*Adv. Funct. Mater.* **2021**, *31*, 2106491
laser-induced graphene	100	10 mV s^{-1}	3.7 mF cm^{-2} at 10µA cm^{-2}	4.5 µWh cm^{-2}	90.5 µW cm^{-2}	75% - 3,200	*Electrochim. Acta*, **2020,** *357*, 136838
GO	100	50 mV s^{-1}	96.2 mF cm^{-2} at 0.2 mA cm^{-2}	8.55 µWh cm^{-2}	0.0853 mW cm^{-2}	75.3% - 3,000	*Nano Energy*, **2019,** *64*, 103938
Graphite	60	100 mV s^{-1}	9.1 mF at 50 mV s^{-1}	/	/	72%-500	*J. Mater. Chem. A*, **2022**, *10*, 12900
Au-CNT	100	1 V s^{-1}	13 mF cm^{-2} at 50 mV s^{-1}	9.6 µWh cm^{-2}	/	90%-10,000	*Adv. Energy Mater.*, **2021,** *11*, 2101523
PPY/CNT	80	50 mV s^{-1}	302 mF cm^{-2} at 1 mA cm^{-2}	10 µWh cm^{-2} (25 °C)	0.6 mW cm^{-2} (25 °C)	87%-60	*Carbon Energy.*, **2022**, *4*, 527
$Li_4Ti_5O_{12}$//activated graphene	80	10 mV s^{-1}	28.4 mF cm^{-2} at 0.05 mA cm^{-2}	39.6 Wh cm^{-3}	2.3 W cm^{-3}	88%-2,000	*Energy Environ. Sci.*, **2018**, *11*, 2001
BCN nanotube	100	100 mV s^{-1}	37.6 mF cm^{-2} at 10 mA cm^{-2}	1.3 mWh cm^{-3}	24.9 mW cm^{-3}	86.1% -200	*Small*, **2021**, *17*, 2102899
Graphene	80	50 mV s^{-1}	362.6 F g^{-1} at 1A g^{-1}	144.9 Wh kg^{-1}	179.2 W kg^{-1}	77%-10,000	*Chem. Eng. J*, **2023**, *451*, 138512

PANI	80	40 mV s^{-1}	87.5 mF cm^{-2} at 1 mA cm^{-2}	8.5 μWh cm^{-2} (25°C)	80 μW cm^{-2} (25°C)	98%-10,000	*Adv. Funct. Mater.*, **2022,** *32*, 2205708
SiC NWs arrays	150	200 mV s^{-1}	18.5 mF cm^{-2} at 2 mA cm^{-2}	/	/	80% - 10,000	*Adv. Funct. Mater.* **2021,** *31*, 2008901
NiO	60	20 mV s^{-1}	18 mF cm^{-2} at 0.1 mA cm^{-2}	/	/	80%-10,000 (25 °C)	*ACS Appl. Mater. Interfaces*，**2020,** *12*, 51978
N-3DG/CNT	70	30 mV s^{-1}	79.8 F cm^{-3} at 30 mV s^{-1}	78.88 mW cm^{-3}	1.65 mW cm^{-3}	81.1%-1,000	*Small*, **2022**, *18*, 2203166
GaN nanocrystalline	150	50 V s^{-1}	52.58 mF cm^{-2} at 0.8 mA cm^{-2}	11.1 μWh cm^{-2}	13.5 mW cm^{-2}	86.2% -10,000	*J. Mater. Chem. A* **2022**, *10*, 22007
RGO Ionogel	150	50 V s^{-1}	0.23 mF cm^{-2}	12.4 μWh cm^{-2}	/	/	*Small*, **2022**, *18*, 2200916
$Ti_3C_2T_x$/ANF	80	50 mV s^{-1}	160 F cm^{-3} at 1 mA cm^{-3}	15 mWh cm^{-3}	0.34 W cm^{-3}	91%-10,000 (25 °C)	*Adv. Funct. Mater.*, **2021**, *31*, 2010944
carbon	82	10 mV s^{-1}	217 F g^{-1} at 0.2 A g^{-1}	100 Wh kg^{-1}	1 KW kg^{-1}	87.5%-20	*J. Mater. Chem. A*, **2021**, *9*, 2714
SiC/CNT	50	50 mV s^{-1}	9.76 F g^{-1} at 0.04 A g^{-1}	1.23 Wh kg^{-1} (25 °C)	32.63 KW kg^{-1} (25 °C)	89.3%-10,000	*J. Mater. Chem. A*, **2022**, *10*, 15708
AC	60	50 mV s^{-1}	270 F g^{-1}	138.6 W h kg^{-1}	18 KW kg^{-1}	80%-5,500	*Nano Energy*, **2021,** *90*, 106500

Table S4. The bader charges corresponding to atoms of GaN/NCO heterostrusture, $NiCoO_2$ surface, GaN surface

	Heterostructure GaN/NCO					NCO			GaN	
	Ni	Co	O	N	Ga	Ni	Co	O	N	Ga
Bader (|e|)	0.96	2.16	-0.90	0.92	1.05	0.73	1.95	-0.79	-1.20	0.92

References

[1] G. Kresse, J. Furthmüller, Phys. Rev. B, 1996, 54, 11169.

[2] G. Kresse, D. Joubert, Phys. Rev. B, 1999, 59, 1758.

[3] J. P. Perdew, K. Burke, M. Ernzerhof, Phys. Rev. Lett., 1996, 77, 3865.

[4] J. Heyd, G. E. Scuseria, M. Ernzerhof, J. Chem. Phys., 2003,118, 8207.

[5] A. V. Krukau, O. A. Vydrov, A. F. Izmaylov, G. E. Scuseria, J. Chem. Phys., 2006, 125, 224106.
